# A novel hood with a small-caliber tip and two traction strings for endoscopic submucosal dissection

**DOI:** 10.1055/a-2239-8666

**Published:** 2024-02-02

**Authors:** Kinya Fujita, Yutaka Saito

**Affiliations:** 1 38607 Gastroenterology, Kobe Adventist Hospital, Kobe, Japan; 2 Endoscopy Division, National Cancer Center Hospital, Tokyo, Japan

Traction techniques have been reported to be useful for performing endoscopic submucosal dissection (ESD) safely 1 2 . We previously reported on use of the Dual Traction (DT) hood with two traction strings installed for performing ESD ( Fig. 1 ) 3 4 5 . Herein, we report an improved DT hood with an attached small-caliber tip that we created, the DT hood type-S ( Fig. 2 ).

**Fig. 1:**
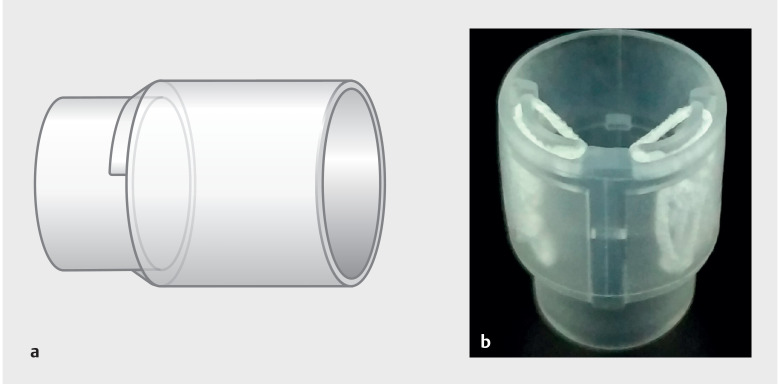
The Dual Traction (DT) hood contains two elastic traction strings in its side pockets.

**Fig. 2:**
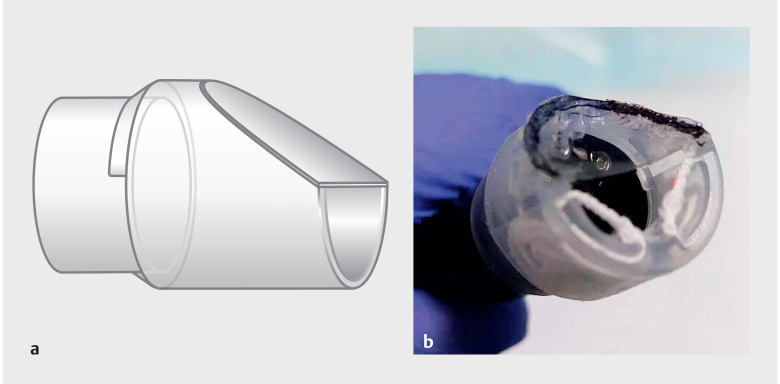
The DT hood type-S used in this report is an improved type of DT hood with an attached small-caliber tip, along with the two elastic traction strings.

An 81-year-old man underwent total colonoscopy for abdominal discomfort, which revealed a laterally spreading tumor nongranular type (LST-NG) of 25 mm in diameter at the cecum ( Fig. 3 **a** ). After informed consent had been obtained, ESD was performed using a colonoscope (PCF-Q260AZI; Olympus, Japan) and the novel hood. With the benefit of the small-caliber tip, the initial mucosal flap was safely created at the anal side using a DualKnife (1.5 mm; Olympus, Japan) and VIO300D (ERBE, AMCO Inc., Japan) ( Fig. 3 **b** ; Video 1 ). After a circumferential mucosal incision had been completed, the first point of traction was applied to the mucosal flap using the traction string. In addition, we applied a second point of traction using the second string to get better visibility of the submucosal layer ( Fig. 3 **c,d** ; Video 1 ). After creating the double traction, we safely performed en bloc resection, without any adverse events ( Fig. 3 **e** ). Histopathologic findings of the resected specimen showed an intramucosal well-differentiated tubular adenocarcinoma, without vascular or lymphatic invasion ( Fig. 4 ).

**Fig. 3:**
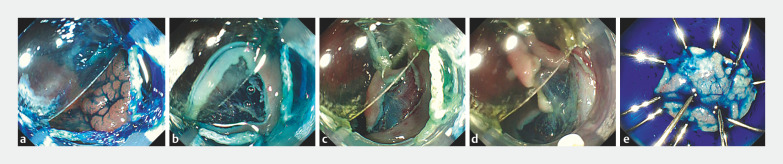
Images from a total colonoscopy showing: **a** a laterally spreading tumor nongranular type of 25 mm in diameter at the cecum, suggestive of an adenomatous lesion; **b** the small-caliber tip of the novel hood helping to easily and safely create the initial mucosal flap (IMF) in front of the lesion; **c** after completion of a circumferential incision of the mucosal layer, the first traction applied to the IMF using the traction string of the novel hood; **d** the second traction applied using the second string to gain better vision of the submucosal layer; **e** the en bloc specimen resected by double traction, without any adverse events.

The small-caliber tip and two traction strings of the novel hood contribute, respectively, to ease of creation of an initial mucosal flap and to better visualization of the submucosal layer than with single traction.Video 1

**Fig. 4:**
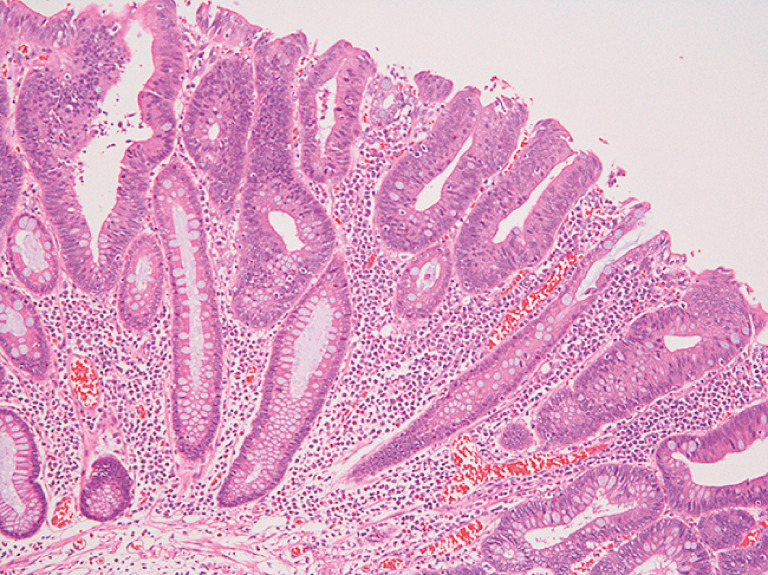
Histopathologic appearance of the resected specimen showing an intramucosal well-differentiated adenocarcinoma, without vascular or lymphatic invasion, and with a negative cut margin.

There are two known difficulties in performing ESD. The first is safely creating the initial mucosal flap for entry into the submucosal layer. The second is maintaining good visibility of the submucosal layer during the entire submucosal dissection. When using the novel hood, the small-caliber tip safely creates the initial mucosal flap and the double traction contributes to improved direct visibility of the submucosal layer compared with conventional single traction ( Fig. 5 ).

**Fig. 5:**
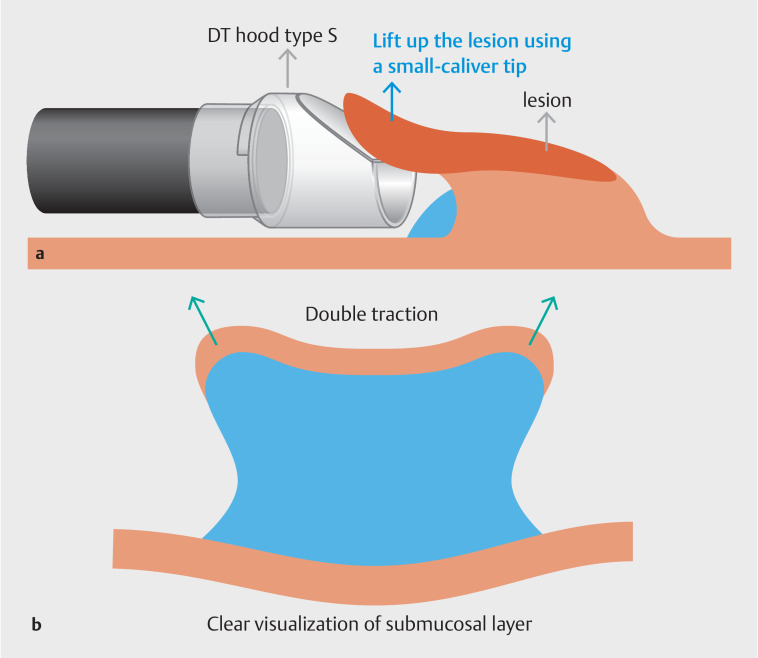
Schematic showing: **a** the small-caliber tip of the novel hood helping to safely create the initial mucosal flap; **b** double traction from the two traction strings of the present hood, which contributes to improved direct visibility of the submucosal layer compared with conventional single traction.

A limitation of this case is that there is room for improvement in the endoscopic view because of the self-made hood; however, improvement is expected with future modifications of the device.

Endoscopy_UCTN_Code_TTT_1AQ_2AD

## Notes

Endoscopy E-Videos https://eref.thieme.de/e-videos
*E-Videos* is an open access online section of the journal *Endoscopy* , reporting on interesting cases and new techniques in gastroenterological endoscopy. All papers include a high-quality video and are published with a Creative Commons CC-BY license. Endoscopy E-Videos qualify for HINARI discounts and waivers and eligibility is automatically checked during the submission process. We grant 100% waivers to articles whose corresponding authors are based in Group A countries and 50% waivers to those who are based in Group B countries as classified by Research4Life (see: https://www.research4life.org/access/eligibility/ ). This section has its own submission website at https://mc.manuscriptcentral.com/e-videos .
